# Read the Journals

**Published:** 1916-11

**Authors:** 


### Read the Journals.

This is distinctly the era of periodical literature. Some people read books, and books will always be read, but the busy man will read ten times as much periodical literature as he will read in books. This is true also of the professions, especially of the medical profession. What busy doctor, with the incessant demands upon him and interruptions to which he is constantly subjected, can take the time to pore over the cumbersome books that are written upon the hundreds of subjects of interest to the profession? The physician must keep up, and about all that he can do is to read the brief discussions that are found in the medical journals, referring to the big text-books only when he finds that a necessity. Whether this is best or not need not be discussed, but it is true that it is the almost universal custom among successful physicians to try to keep up with the current problems as set out in the periodicals, and they are forced to do this almost to the exclusion of the bookish discussions. Of course, a few physicians do not read much of anything, but they are a negligible quantity so far as professional progress goes.

Another reason why the periodical has become so popular is that many diseases become prevalent and then subside before books treating of them can be published. A few weeks ago the whole country was wrought up over infantile paralysis, and there was great fear that it would spread all over America, but it appears to have been checked almost as quickly as it came. The journals of the country discussed it freely then, but books on the subject are still in the making. The great advantage of the periodical is that it handles issues while they are live, and handles them in such a pointed way that the busy practitioner has the time to keep informed. The physician or surgeon who boasts that he hasn't the time to read medical journals is boasting of his lack of information upon the things in his profession which everyone should know.

